# Priming of the murine mammary gland with *Staphylococcus chromogenes* IM reduces bacterial growth of *Streptococcus uberis*: a proof-of-concept study

**DOI:** 10.1186/s13567-023-01156-y

**Published:** 2023-03-27

**Authors:** Niels Vander Elst, Julie Bellemans, Jonas Steenbrugge, Chloë Geeroms, Koen Breyne, Sofie Piepers, Bruno Toledo-Silva, Fernando Nogueira de Souza, Freddy Haesebrouck, Sarne De Vliegher, Evelyne Meyer

**Affiliations:** 1 Laboratory of Biochemistry, Department of Veterinary and Biosciences, Faculty of Veterinary Medicine, Ghent University, Merelbeke, Belgium; 2 M-Team and Mastitis and Milk Quality Research Unit, Department of Internal Medicine, Reproduction and Population Medicine, Faculty of Veterinary Medicine, Ghent University, Merelbeke, Belgium; 3 Molecular Neurogenetics Unit, Neurology and Radiology Department, Massachusetts General Hospital-Harvard Medical School, 13th Street, Building 149, Charlestown, MA 02129 USA; 4 Department of Internal Medicine, Faculty of Veterinary Medicine and Animal Science, University of São Paulo, São Paulo, Brazil; 5 Department of Pathobiology, Pharmacology and Zoological Medicine, Faculty of Veterinary Medicine, Ghent University, Merelbeke, Belgium

**Keywords:** Non-*aureus* staphylococci, priming, *Streptococcus uberis*, *Staphylococcus chromogenes*, bovine mastitis, dairy cow

## Abstract

*Streptococcus uberis* is a major causative agent of bovine mastitis, an inflammation of the mammary gland with substantial economic consequences. To reduce antibiotic use in animal agriculture, alternative strategies to treat or prevent mastitis are being investigated. Bovine-associated non-*aureus* staphylococci are proposed in that respect due to their capacity to inhibit the in vitro growth of *S. uberis*. We demonstrate that priming the murine mammary gland with *Staphylococcus chromogenes* IM reduces *S. uberis* growth in comparison with non-primed glands. The innate immune system is activated by increasing IL-8 and LCN2, which may explain this decreased growth.

## Introduction, methods and results

Bovine mastitis is defined as the inflammatory response of the mammary gland of cows in response to an intramammary infection, mostly by bacteria. As it is such a prevalent infectious disease in dairy cows, it causes major economic losses to the global dairy industry. Also, bovine mastitis impacts farmers serenity, leads to substantial food waste and threatens cow welfare. In view of the emerging antimicrobial resistance crisis, there is a sense of urgency to reduce the use of antimicrobials, also in the livestock sector [1]. Alternatives to small molecule antibiotics dominating the bovine mastitis field are warranted [2]. Hence, solutions might be found in bacteria adapted to the mammary gland niche, such as some non-*aureus* staphylococci (NAS) [2]. Historically, NAS are considered minor mastitis pathogens, but emerging reports claim that some of the NAS are relevant concerning udder homeostasis [2, 3]. *Staphylococcus chromogenes* is the most prevalent NAS species and has been retrieved from bovine teat skin, used bedding and bulk tank milk, classifying it as a host-adapted bacterium [4]. Still, differences in adherence to bovine mammary epithelial cells between *S. chromogenes* strains have been demonstrated in vitro [5], as well as differences in the colonization of the mammary gland in mice [6] and in heifers [7]. Protective effects of *S. chromogenes* against intramammary infection with major pathogens such as *Staphylococcus aureus* and *Streptococcus uberis* have been described [8–10]. These effects have been related to bacterial secreted mediators such as bacteriocins and other potentially beneficial mechanisms such as priming of the immune system [9, 11]. We previously showed that *S. chromogenes* IM, a bovine-derived mastitis strain, is able to (i) inhibit the in vitro growth of major mastitis pathogens including *S. uberis* and (ii) colonize the bovine mammary gland in vivo [12, 13]. To better understand whether priming the mammary gland with *S. chromogenes* IM inhibits the growth of *S. uberis*, mouse experiments were hereafter conducted in which *S. chromogenes* IM-primed murine mammary glands were challenged with *S. uberis*. Our groups have previously demonstrated such growth inhibition of *S. aureus* by priming the murine mammary gland with lipopolysaccharides and lipoteichoic acids [14]. The relevance and comparison of the mouse as a mastitis model for dairy cows was previously described by our groups [15].

**Figure 1:**
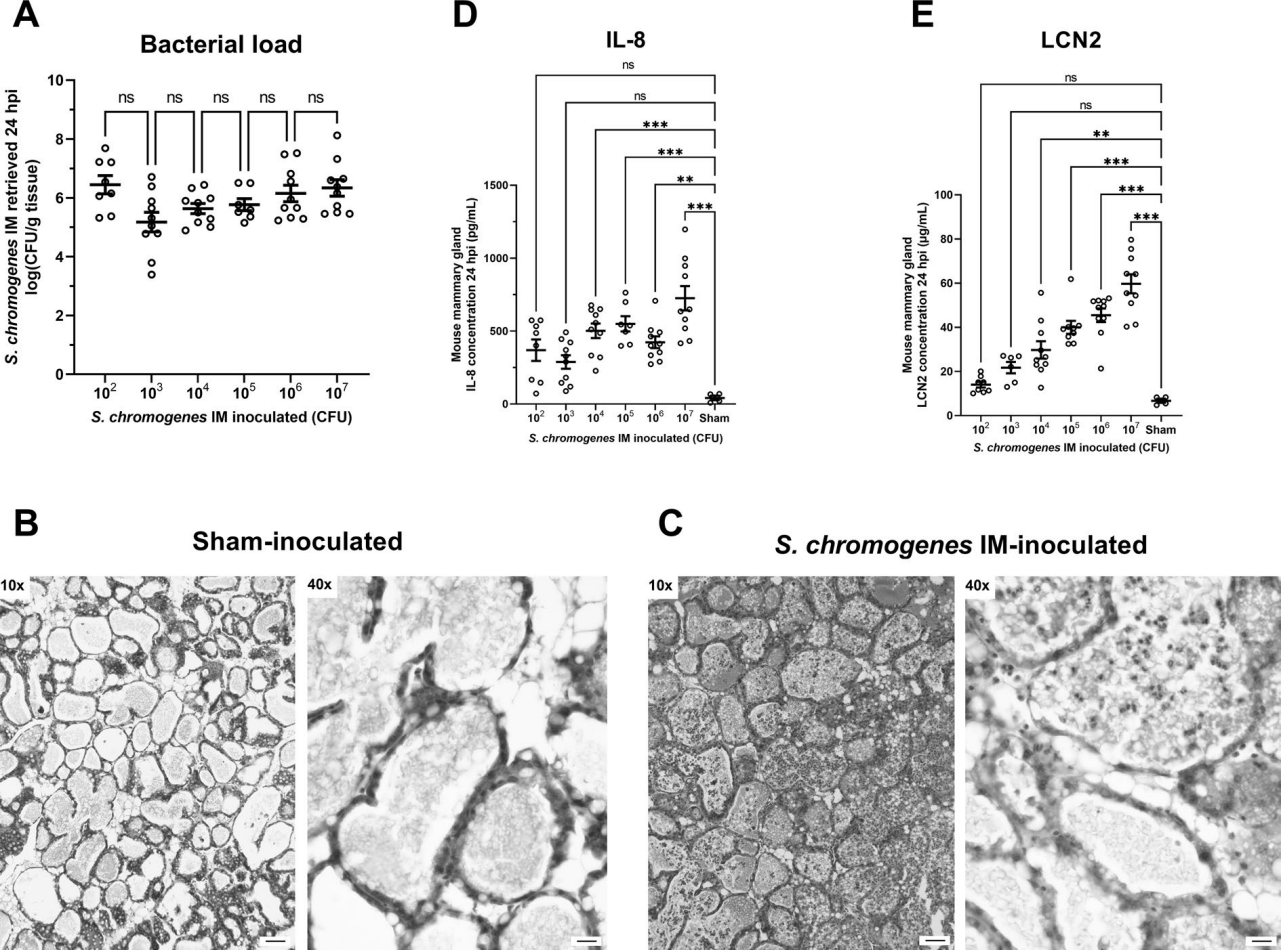
**Murine mammary gland response to inoculation with different doses of Staphylococcus chromogenes IM. A** Bacterial load, **D** IL-8 and **E** LCN2 concentrations in mouse mammary glands harvested 24 h after intraductally inoculating *Staphylococcus chromogenes* IM at serial doses ranging from 10^2^ to 10^7^ CFU, as well as representative H&E-stained sections of **B** sham (PBS) or **C** *S. chromogenes* IM-inoculated mammary glands (10^4^ CFU) at 24 h post-inoculation. Data are shown as individual points with a line indicating the mean and an error bar representing the standard error of the mean (SEM). Double (**) and triple (***) asterisks indicate respectively *p* < 0.01 and *p* < 0.001, whereas “ns” indicates non-significance corresponding to a *p* > 0.01. Pictures were taken at a 10 × and 40 × magnification, with scale bars indicating 100 µm and 25 µm, respectively.

Firstly, the optimal inoculation dose to study the potential protective effect of *S. chromogenes* IM was determined by characterizing its bacterial growth in the murine mammary gland. For this purpose, lactating Hsd:ICR (CD1) mice were assigned to six different groups and intramammary inoculated in the fourth gland pair with *S. chromogenes* IM using serial doses ranging from 10^2^ to 10^7^ colony-forming units (CFU) (number of animals; n_animals_ = between 8 and 10 per dose group). A seventh group received sham (phosphate-buffered saline, PBS) as a negative control (n_animals_ = 6). We refer to our previous papers for a detailed methodology of inoculum preparation, intraductal inoculation, euthanasia of the mice, tissue harvesting and processing [6, 14, 16]. Data were analysed using GraphPad Prism (version 9.5.0) to calculate *P*-values and determine statistically significant differences (*P* < 0.05). Two groups were compared with unpaired, two-tailed t-tests, and multiple groups with analysis of the variance (ANOVA) and a Bonferroni post hoc-test. Mice were clinically scored from 0 to 3 based on body weight and temperature, body condition score, behaviour and general appearance at 24 h post-inoculation (hpi) inspired by the Morton and Griffiths scheme (1985). This clinical score was comparable for the *S. chromogenes* IM- and sham-inoculated animals, i.e., 0.38 ± 0.18 (n_animals_ = 8) and 0.17 ± 0.17 (n_animals_ = 6), respectively (mean ± standard error of the mean (SEM); data provided are for the group with an intermediate inoculum dose of 10^4^ CFU). Upon necropsy, mice inoculated with *S. chromogenes* IM showed swollen mammary glands with increased vascularization and redness compared to the sham-inoculated group. However, no differences were macroscopically observed between the six *S. chromogenes* IM inoculum dose groups. Corroborating this qualitative observation, *S. chromogenes* IM attained a similar bacterial load in all inoculated groups at 24 hpi (Figure 1A). Inoculated mammary glands were also evaluated microscopically. The luminal area was filled with milk in the sham-inoculated animals, while in all six *S. chromogenes* IM-inoculated groups the presence of luminal immune cells was observed (Figures 1B, C). The majority of these were identified as polymorphonuclear cells (PMNs) based on their characteristic multilocular feature (Figure 1C). To further evaluate the observed innate immune response to *S. chromogenes* IM, concentrations of the pro-inflammatory mediators interleukin (IL)-8 (i.e., the murine analogue MIP-2) and neutrophil gelatinase-associated lipocalin (LCN)2 (a.k.a. NGAL) were determined on mammary gland lysates with validated immuno-assays as previously described by our group [6, 14, 16]. For both IL-8 and LCN2, an inflection point was observed for the 10^4^ CFU inoculation dose (Figures 1D, E). This means that the IL-8 and LCN2 concentrations for the 10^4^, 10^5^, 10^6^ and 10^7^ CFU inoculated animals differed significantly (*p* < 0.01) from the sham group (Figures 1D, E). In contrast, IL-8 and LCN2 concentrations for mice inoculated with 10^2^ and 10^3^ *S. chromogenes* IM did not differ significantly (*p* > 0.01) from sham-inoculated mice (Figures 1D, E). Interestingly, the concentration of LCN2 followed an increasing trend correlating with the inoculation dose (Figure 1E). Following this inflection point at 10^4^ CFU, it was decided to proceed in the *S. chromogenes IM/S. uberis* superchallenge experiment with the latter dose to investigate if the observed priming of the mammary gland immune system by *S. chromogenes* IM was sufficient to prevent *S. uberis* growth.

**Figure 2:**
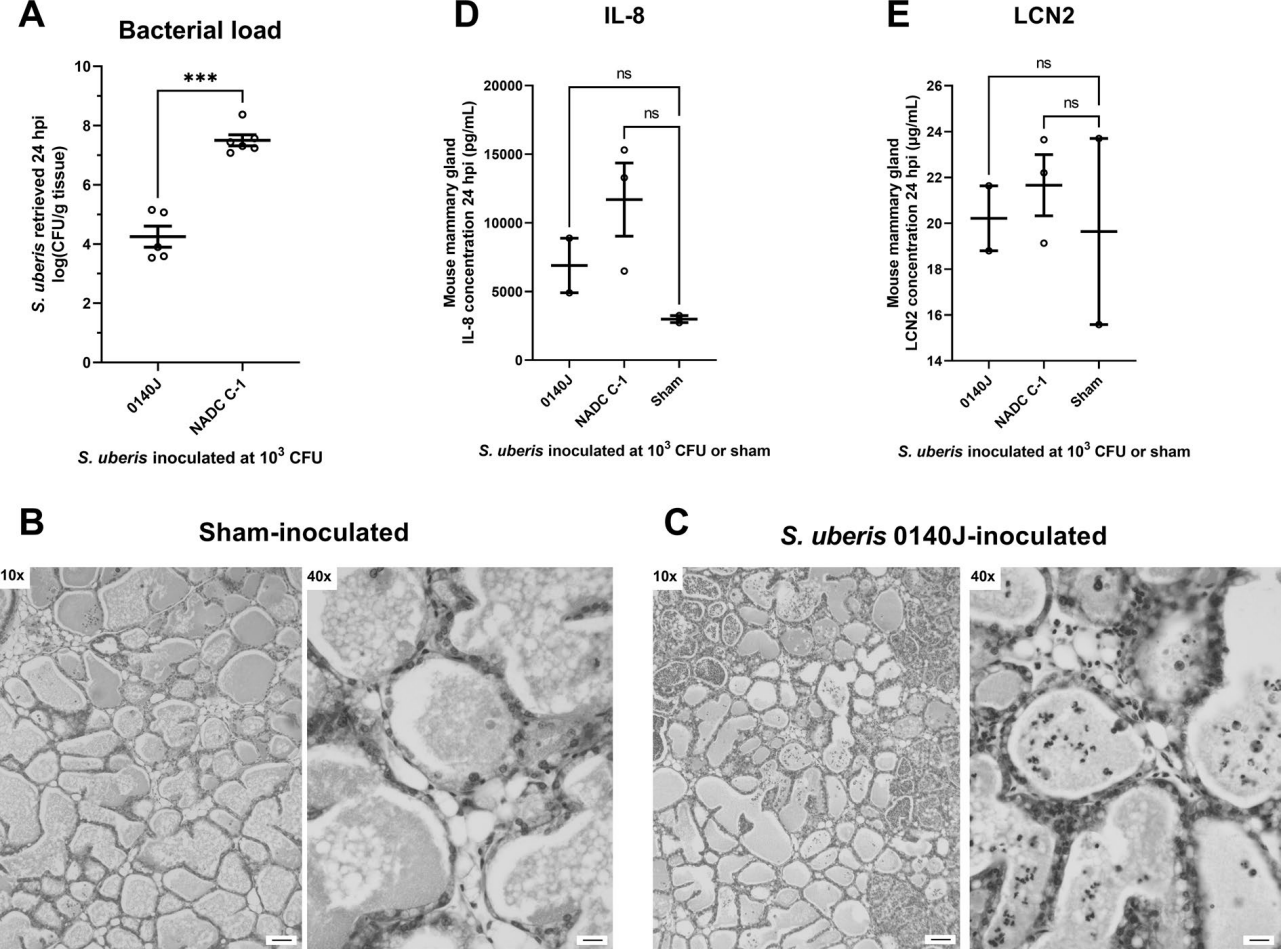
**Murine mammary gland response to inoculation with different strains of Streptococcus uberis. A** Bacterial load, **D** IL-8 and **E** LCN2 concentrations in mouse mammary glands harvested 24 h after intraductally inoculating 10^3^ CFU *S. uberis* 0140J or NADC C-1, as well as representative H&E-stained sections of **B** sham or **C** *Streptococcus uberis* 0140J-inoculated mammary glands at 24 h post-inoculation. No CFUs were retrieved from the *S. uberis* NCTC 3858 inoculated mouse mammary glands. Data are shown as individual points with a line indicating the mean and an error bar representing the SEM. A triple (***) asterisk indicates *p* < 0.001, whereas “ns” indicates non-significance corresponding to a *p* > 0.01. Pictures were taken at a 10 × and 40 × magnification, with scale bars indicating 100 µm and 25 µm, respectively.

Secondly, an *S. uberis* infection experiment was performed to identify a suitable strain able to efficiently colonize the murine mammary gland. Three bovine-derived *S. uberis* ATCC strains, i.e., ATCC BAA-854 (a.k.a. 0140J), ATCC 27958 (a.k.a. NADC C-1) and ATCC 19436 (a.k.a. NCTC 3858) were intramammary injected with an inoculum of 10^3^ CFU to evaluate the same parameters as selected for *S. chromogenes* IM at 24 hpi, in comparison with sham-inoculated glands again used as a negative control. The clinical scores assigned to the inoculated mice were highest in the *S. uberis* NADC C-1 group, followed by the 0140J and NCTC 3858 groups, and were lowest in the sham-inoculated group i.e., 1.00 ± 0.41 (n_animals_ = 4), 0.75 ± 0.25 (n_animals_ = 4), 0.50 ± 0.50 (n_animals_ = 2) and 0.33 ± 0.33 (n_animals_ = 3), respectively. Upon necropsy, the glands inoculated with the *S. uberis* 0140J and NADC C-1 strains showed similar macroscopic signs as the *S. chromogenes* IM inoculated glands, i.e., swelling, vascularization and redness, in marked contrast to the *S. uberis* NCTC 3858-inoculated glands which were comparable to the sham-inoculated glands. Corroborating these qualitative findings, a bacterial load of 4.25 ± 0.36 (n_glands_ = 5) and 7.50 ± 0.19 (n _glands_ = 6) log CFU/g tissue was retrieved from the *S. uberis* 0140J and NADC C-1 inoculated glands, respectively (Figure 2A). Again, in marked contrast, *S. uberis* was not isolated from mouse mammary glands inoculated with strain NCTC 3858, supporting the macroscopic observations. Therefore, *S. uberis* NCTC 3858 was excluded as a candidate strain for the final *S. chromogenes IM/S. uberis* superchallenge experiment. Upon the subsequent microscopic evaluation, an influx of immune cells in the alveoli was observed in the *S. uberis* 0140J and NADC C-1 inoculated mammary glands which were again absent in the sham-inoculated glands (Figures 2B, C). The influx and morphology of these immune cells were similar to that in *S. chromogenes* IM-challenged glands, identifying them as PMNs based on their polymorphonuclear feature. The PMN influx was not homogeneous across the murine mammary gland, in the sense that not every duct or gland contained the same amount of PMNs (Figure 2C). The IL-8 concentrations non-significantly increased (*p* > 0.01) in the *S. uberis* 0140J and NADC C-1 inoculated glands versus (vs.) the negative control (Figure 2D). Likewise, no significant difference (*p* > 0.01) was seen in LCN2 concentrations between the *S. uberis* 0140J and NADC C-1 inoculated glands vs*.* the negative control. Given its significantly (*p* < 0.001) higher and less variable bacterial load in the infected murine mammary glands at 24 hpi (Figure 2A), the *S. uberis* NADC C-1 strain was chosen for the final *S. chromogenes* IM*/S. uberis* superchallenge experiment.

**Figure 3:**
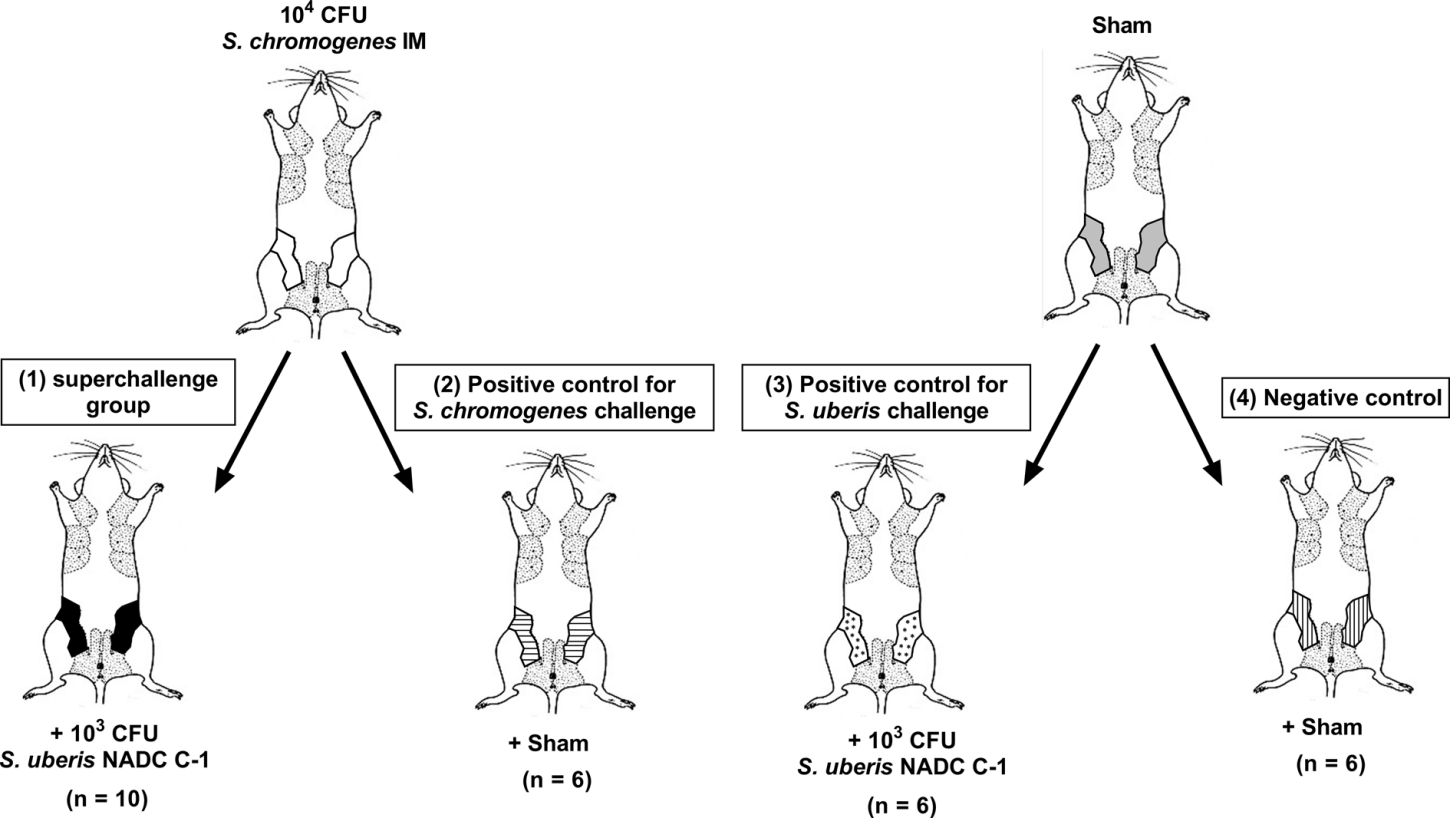
**Representation of the superchallenge experiment to investigate Staphylococcus chromogenes priming against Streptococcus uberis infection.** Mice were intramammarily inoculated with either 10^4^ CFU *S. chromogenes* IM (challenge) or sham (PBS), followed by a second inoculation 24 h later with either 10^3^ CFU *S. uberis* NADC C-1 (infection) or sham (PBS). This led to 4 groups i.e., (1) *S. chromogenes* IM-*S. uberis*-inoculated (black), (2) *S. chromogenes* IM-sham (horizontal stripes), (3) sham-*S. uberis* (asterisks) and (4) sham-sham (vertical stripes) mice. “n” represents the number of animals inoculated in each group.

**Figure 4:**
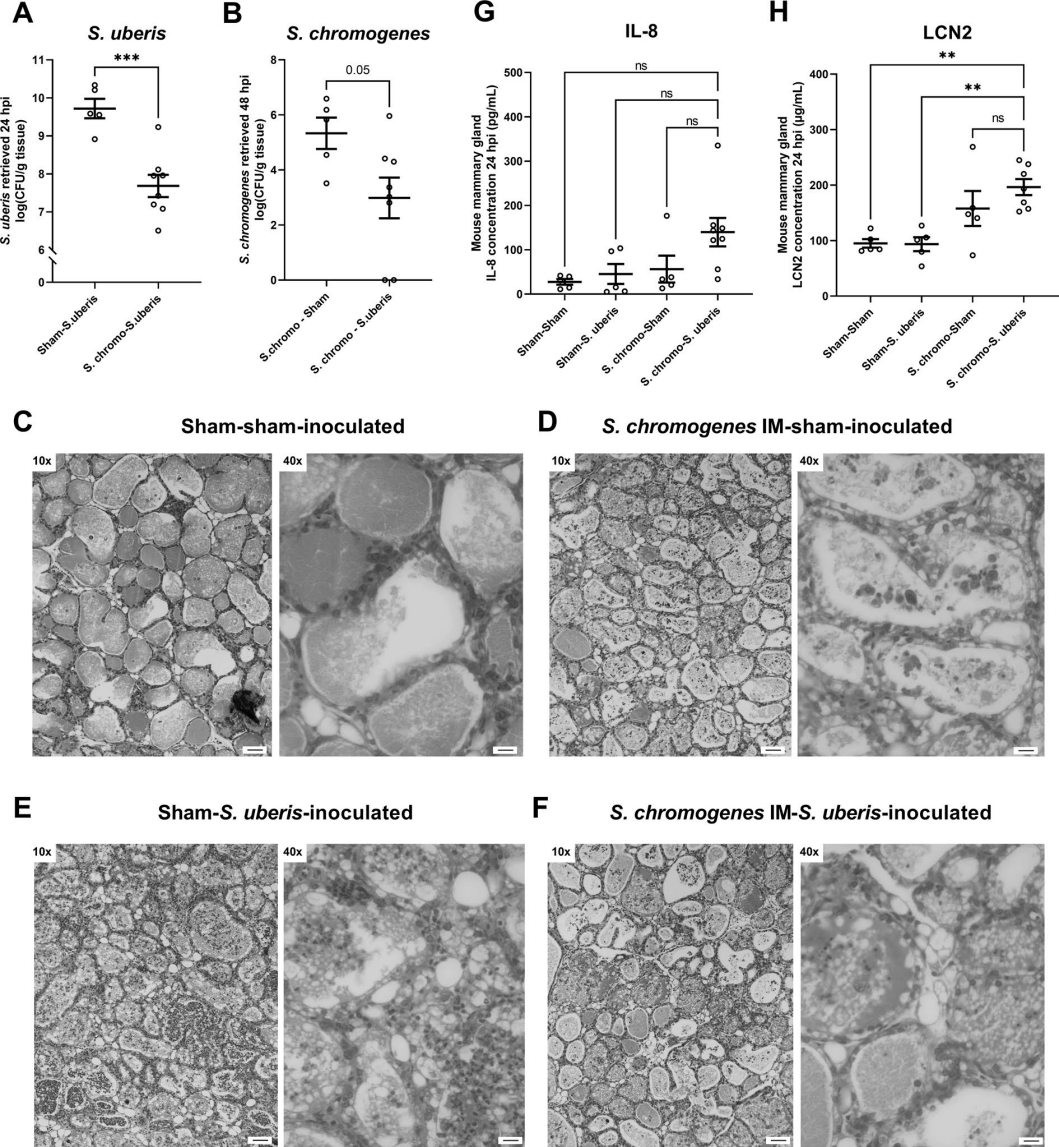
**Priming effect of Staphylococcus chromogenes IM in the murine mammary gland against Streptococcus uberis infection.** Bacterial growth of **A** *S. uberis* and **B** *S. chromogenes* IM, concentrations of **G** IL-8 and **H** LCN2 in mouse mammary glands, as well as representative H&E-stained sections thereof, harvested 48 h after intraductally inoculating** C**, **E** sham (PBS) or **D**, **F** 10^4^ CFU *S. chromogenes* IM, followed by a second inoculation after a 24 h interval with either **C**, **D** sham or **E**, **F** 10^3^ CFU *S. uberis* NADC C-1. Data are shown as individual points with a line indicating the mean and an error bar representing the SEM. Double (**) and triple (***) asterisks indicate respectively *p* < 0.01 and *p* < 0.001, whereas “ns” indicates non-significance corresponding to a *p* > 0.05. Pictures were taken at a ×10 and ×40 magnification, with scale bars indicating 100 µm and 25 µm, respectively.

Thirdly, the hypothesized protective effect of a priming with *S. chromogenes* IM against *S. uberis* NADC C-1 was investigated as follows (schematically shown in Figure 3): (1) a first group of mice received 10^4^ CFU *S. chromogenes* IM followed by challenge with 10^3^ CFU *S. uberis* NADC C-1 24 h later (i.e., the so-called superchallenge group), (2) a second group received a sham (PBS) inoculation following the 10^4^ CFU *S. chromogenes* IM inoculation administered 24 h earlier (i.e., the positive control for *S. chromogenes* IM challenge), (3) a third group also received a sham (PBS) inoculation but this now preceded the *S. uberis* NADC C-1 challenge administered 24 h later (i.e., the positive control for *S. uberis* infection) and (4) a fourth group twice received a sham (PBS) inoculation (i.e., the negative control). At 48 h, i.e., 24 h after these second inoculations, all mice were euthanized and their mammary glands were harvested and processed as described before. The clinical score assigned to the four groups did not differ, i.e., 1.78 ± 0.15 (n_animals_ = 9) for *S. chromogenes* IM-*S. uberis* NADC C-1 (superchallenge group), 1.83 ± 0.31 (n_animals_ = 6) for *S. chromogenes* IM-sham, 1.83 ± 0.17 (n_animals_ = 6) for sham-*S. uberis* NADC C-1 and 1.83 ± 0.31 (n_animals_ = 6) for sham-sham. Macroscopic evaluation of the glands indicated swelling, increased vascularization and redness in all groups, regardless of the inoculum. A key finding, however, was the significant reduction (*p* < 0.001) of the *S. uberis* NADC C-1 load in the superchallenge group of 2.04 ± 0.43 log (i.e., 99%; difference between means ± SEM) compared to the sham-*S. uberis* NADC C-1 group (Figure 4A). On the other hand, *S. chromogenes* IM also showed a reduced (*p* = 0.05) bacterial growth of 2.35 ± 1.05 in the superchallenge group vs*.* the *S. chromogenes* IM-sham group (Figure 4B). As expected, no bacteria were isolated from the sham-sham inoculated group. Confirmation of the retrieved bacterial species after plating serial dilutions of the mammary gland homogenates on Columbia blood agar (5% defibrinated sheep blood) was done by MALDI-ToF MS as described by our group (data not shown) [13]. Plating on this agar allowed to distinguish *S. uberis* from *S. chromogenes* based on colony morphology. Microscopic evaluation of the mammary glands showed that the sham-*S. uberis* NADC C-1, *S. chromogenes* IM-sham and *S. chromogenes* IM-*S. uberis* NADC C-1 groups all induced a similar influx of PMNs in the alveoli, which was again absent in the sham-sham inoculated glands (Figures 4C–F). The IL-8 concentrations in the *S. chromogenes* IM*-S. uberis* NADC C-1-treated mammary gland lysates were non-significantly augmented (*p* > 0.05) in comparison with the sham-sham, sham-*S. uberis* and *S. chromogenes* IM-sham inoculated glands (Figure 4G). As for the LCN2 concentrations, the *S. chromogenes* IM-*S. uberis* NADC C-1 gland lysates differed significantly (*p* < 0.01) from both non-primed groups, i.e. sham-sham and sham-*S. uberis*, but not from the *S. chromogenes* IM-sham inoculated glands (*p* > 0.05) (Figure 4H).

## Discussion

Mastitis is the most common disease in modern dairy farming, causing economic losses and resulting in substantial antibiotic use [17, 18]. Given the society demand as well as governmental policies (e.g., European Green Deal, WHO; new EU Regulation on Veterinary Medicines 2019/06) to reduce antimicrobial use in animal agriculture, interest in alternative strategies such as the use of probiotics has become very actual [18]. In the specific case of bovine mastitis, recent insights are suggesting the use of bovine NAS to prime the mammary gland and prevent infection by major mastitis pathogens [8, 9, 11]. In this proof-of-concept study, priming of the mammary gland with *S. chromogenes* IM, a NAS strain previously characterized and used by our groups in multiple in vitro and in vivo studies [5–7, 12, 13, 19], was investigated to counteract mastitis caused by the major mastitis pathogen *S. uberis* in a mouse mastitis model [14, 15]. First, the inoculum dose and priming potential of the murine mammary gland by this specific NAS strain were studied. It was observed that *S. chromogenes* IM attained a similar bacterial load in the murine mammary gland regardless of the inoculation dose. We hypothesize that a low to moderate *S. chromogenes* IM dose induces a similar priming effect as a high inoculum dose. However, it is important to note that our study cannot confirm that different inoculation doses produce similar priming effects and priming by NAS has not yet been fully elucidated in literature. The choice for a 10^4^ CFU inoculation dose of bacteria can be suggested from the observed increase in iron-chelating LCN2 which coincided with the increase in inoculated bacteria. An increase in the LCN2 refers to an activation of the innate immune system. The mammary tissue is prepared for future major pathogenic infections such as in this case *S. uberis* by LCN2 increase. The more LCN2 is released, the more iron will be chelated and the better bacterial growth can be inhibited [16]. Second, an appropriate *S. uberis* candidate strain (i.e., NADC C-1) was selected based on the high and reproducible bacterial load retrieved as well as a clear influx of PMNs 24 hpi. Third, it was shown that after inoculation with *S. chromogenes* IM, this priming reduces the subsequent mammary growth of *S. uberis* NADC C-1 in the murine mammary gland by 99%. It should be emphasized that *S. uberis* NADC C-1 was still able to colonize the murine mammary gland even in the presence of this particular NAS strain. Indeed, it is important to note that the reduced growth of *S. uberis* NADC C-1 was obtained by priming the murine mammary gland with 10^4^ CFU *S. chromogenes* IM, but the use of other bacterial strains or inoculation doses might have resulted in a different outcome. On the other hand, our research also revealed the limits of *S. chromogenes* IM as a potential priming agent against *S. uberis* mastitis. Likewise observed in dairy cows [13], *S. chromogenes* IM induces mastitis in the murine mammary gland. Indeed, the observed influx of polymorphonuclear immune cells and significantly increased levels of proinflammatory mediators IL-8 and LCN2 support this latter statement. Therefore, it might not be advisable to apply *S. chromogenes*-based primers during cow lactation unless a (slight) increase of the somatic cell count due to this priming effect is accepted [20]. However, from a mastitis prevention point-of-view a promising strategy might be to apply NAS rather in the cow's dry-off period [2]. The spread of NAS, adapted to cows, would be limited for milking equipment [21]. Dry-off also marks the critical point in dairy farming since most antibiotics are prophylactically used in an attempt to reduce the intramammary infection risk during this period and clinical mastitis in the next lactation [17, 18]. In our mouse mastitis model, priming of the immune system by *S. chromogenes* IM was clearly observed and defined by hallmark signs of inflammation as was previously seen for lipopolysaccharide and lipoteichoic acid [14]. Our proof-of-concept superchallenge findings corroborate and further support other reports stating some NAS offer a priming potential to prevent mastitis by the major mastitis pathogen *S. uberis* [9, 11]. Preliminary proof hereof has already been delivered some decades ago but also very recently in the context of *S. aureus* [8, 10]. Taken together, we here showed that *S. chromogenes* IM priming of the murine mammary gland reduces bacterial growth of *S. uberis* NADC C-1 substantially.

## Data Availability

The datasets used and/or analysed during the current study are available from the corresponding author on reasonable request.
